# Supplementary material for: Rapid migrations and dynamics of citizen response
Source: R Soc Open Sci. 2019 Mar 6;6(3):181864. doi: 10.1098/rsos.181864 (PMC6458389; doi:10.1098/rsos.181864)
Supplement: Supplementary Information [file rsos181864supp1.pdf]

# Rapid migrations and dynamics of citizen response
Anand Sahasranaman and Henrik Jeldtoft Jensen

## SUPPLEMENTARY INFORMATION (SI): An exploration of the parameter space.

**SI.1: Sensitivity to maximum population limit:**

In our base model, we set the maximum population growth to be 150% of the initial population. Here we explore the dynamics generated by the model for higher and lower population limits: 125% and 200%. Supplementary Table 1 presents the comprehensive parameter set for these simulations.

**Supplementary Table 1. Model Parameters.**

| | | |
|---|---|---|
| Number of Neighborhoods ($M$) | 20 | 20 |
| Number of Resident Agents | 1000 | 1000 |
| Resident Tolerance Level ($\tau_{res}$) | 0.25 | 0.25 |
| Migrant Tolerance Level ($\tau_{mig}$) | 0.75 | 0.75 |
| Maximum population (% initial) | 125% | 200% |
| Migration Rate ($g$) | 0.004, 0.006, 0.01, 0.015, 0.02, 0.03, 0.05 | 0.009, 0.011, 0.015, 0.02, 0.03, 0.04, 0.05 |
| Number of Iterations | 200 | 200 |
| $\beta_{in}$ | 1 | 1 |

We run an ensemble of 30 runs of the entire model for each value of $g$. Supplementary Figure 1 (left) plots the average time to tolerance breach ($T_\tau$) as a function of fraction of residents whose tolerance has ever been breached ($B_f$), while Supplementary Figure 1 (right) plots the evolution of segregation over the longer term for population limit 125%. We find that the non-linear relationship between $T_\tau$ and $B_f$ is replicated as in the original model; however, given that population increase is capped at 25%, the extent of $B_f$ is not as high as the base scenario for higher $g$. We also observe that segregation (CSI) converges for all $g$ as expected in the Schelling framework.

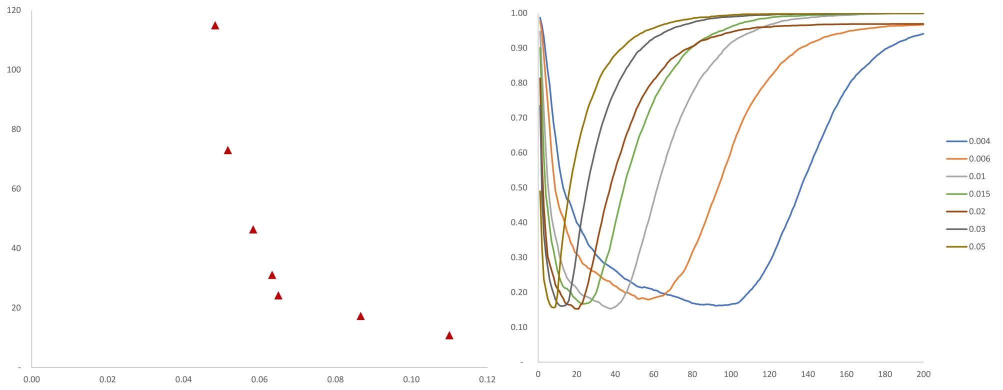


**Supplementary Figure 1.** Sensitivity to Maximum Population Limit (125% of initial population). Left: Average time to tolerance breach ($T_\tau$) v. Total Fraction of residents ever breached ($B_f$). Right: Cell Segregation Indicator (CSI) over time.

Similarly, we find that the model results are replicated even for the higher maximum population limit of 200% of initial population. Supplementary Figure 2 (left) plots the non-linear relationship between $T_\tau$ and $B_f$, and here we find that $B_f$ is higher than the base scenario for higher $g$. Segregation behaviour converges over time (Supplementary Figure 2, right).

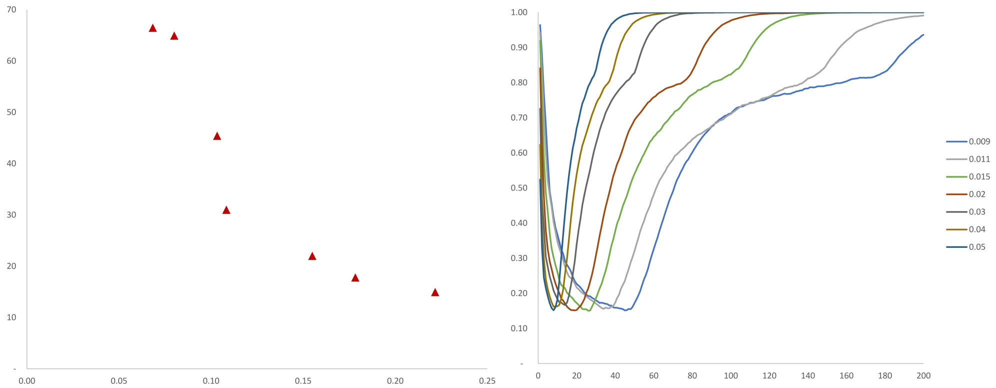


**Supplementary Figure 2.** Sensitivity to Maximum Population Limit (200% of initial population). Left: Average time to tolerance breach ($T_\tau$) v. Total Fraction of residents ever breached ($B_f$). Right: Cell Segregation Indicator (CSI) over time.

**SI.2: Sensitivity to resident tolerance level ($\tau_{\text{res}}$):** Here we explore the dynamics generated by the model for residents with higher tolerance levels than the base model. We use $\tau_{\text{res}} = 0.5$, and test whether the model outcomes are replicated under this scenario. Supplementary Table 2 highlights key model parameters.

**Supplementary Table 2. Model Parameters.**

| | |
|---|---|
| Number of Neighborhoods ($M$) | 20 |
| Number of Resident Agents | 1000 |
| Resident Tolerance Level ($\tau_{\text{res}}$) | 0.5 |
| Migrant Tolerance Level ($\tau_{\text{mig}}$) | 0.75 |
| Maximum population (% initial) | 150% |
| Migration Rate ($g$) | 0.005, 0.007, 0.01, 0.015, 0.02, 0.03, 0.04, 0.05 |
| Number of Iterations | 200 |
| $\beta_{\text{in}}$ | 1 |

We run an ensemble of 30 runs of the entire model for each value of $g$. We find that overall, the behavior of this model is analogous to that of the original model. Supplementary Figure 3 illustrates this similarity - we see a non-linear relationship between $T_\tau$ and $B_f$ (left), and also convergence in long-term segregation behaviour (right). Importantly, we also observe that even with doubling of the tolerance of resident agents, when we compare the relationship between $T_\tau$ and $B_f$ for $\tau_{\text{res}} = 0.5$ (Supplementary Figure 3, left, blue) with the base case of $\tau_{\text{res}} = 0.25$ (Supplementary Figure 3, left, red), the two curves show a remarkable similarity in values attained by $B_f$ and only a slight increase in corresponding $T_\tau$ values. This suggests that increasing $\tau_{\text{res}}$ does not have a significant impact either on both the non-linear nature of the relationship between $B_f$ and $T_\tau$ or on the range of values attained by both $B_f$ and $T_\tau$.

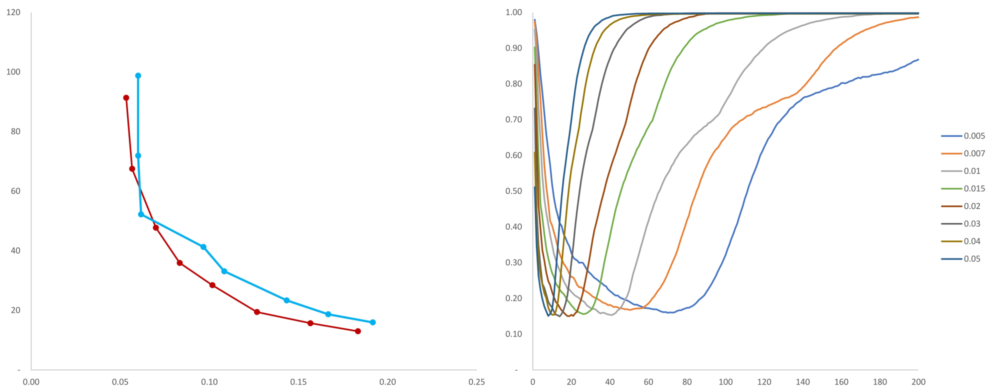


**Supplementary Figure 3.** Sensitivity to resident tolerance level ($\tau_{res}$ = 0.5). Left: Average time to tolerance breach ($T_\tau$) v. Total Fraction of residents ever breached ($B_f$). Blue: $\tau_{res}$ = 0.5. Red: $\tau_{res}$ = 0.25. Right: Cell Segregation Indicator (CSI) over time.

**SI.3: Sensitivity to ease of migrant entry ($\beta_{in}$):**

Supplementary Table 3 presents the complete parameter set for this simulation where we alter the ease of migrant entry into the city. We test the robustness of the results for both higher and lower values of $\beta_{in}$.

**Supplementary Table 3. Model Parameters.**

| | | |
|---|---|---|
| Number of Neighborhoods ($M$) | 20 | 20 |
| Number of Resident Agents | 1000 | 1000 |
| Resident Tolerance Level ($\tau_{res}$) | 0.25 | 0.25 |
| Migrant Tolerance Level ($\tau_{mig}$) | 0.75 | 0.75 |
| Maximum population (% initial) | 150% | 150% |
| Migration Rate ($g$) | 0.012, 0.015, 0.02, 0.03, 0.04, 0.05 | 0.0025, 0.0035, 0.005, 0.0075, 0.01, 0.02, 0.03, 0.05 |
| Number of Iterations | 200 | 200 |
| $\beta_{in}$ | 2 | 0.1 |

Again we run an ensemble of 30 runs of the model for each value of $g$. We find that for $\beta_{in}$ = 10, agent entry into the city is highly unlikely, and over 200 iterations, for $g$ as high as 0.5 and 1.0, we find that $\simeq$ 0.004% of agents attempting entry during the course of simulations were able to actually enter. Therefore, this is essentially a sterile condition with no dynamics of movement. When $\beta_{in}$ = 2, we find that the fraction of agents able to gain entry $\simeq$ 19%, which is lower than our base model where rate of entry was $\simeq$ 43% (as expected), but still generates similar transient and long-term dynamics (Supplementary Figure 4). The non-linear relationship between $T_\tau$ and $B_f$ is apparent (Supplementary Figure 4, left), though given the lower entry rate, we see that the high $B_f$ values are substantially lower than base case.

We also simulate dynamics for $\beta_{in}$ = 0.1, indicating greater ease of entry compared to our base model. We find that under this scenario, on average, $\simeq$ 92% of agents attempting entry are able to do so. Again, we find that the model results are replicated: the non-linear relationship between $T_\tau$ and $B_f$ obtains, with the attainment of higher values for $B_f$; and the convergence of segregation outcomes happens over time (Supplementary Figure 5).

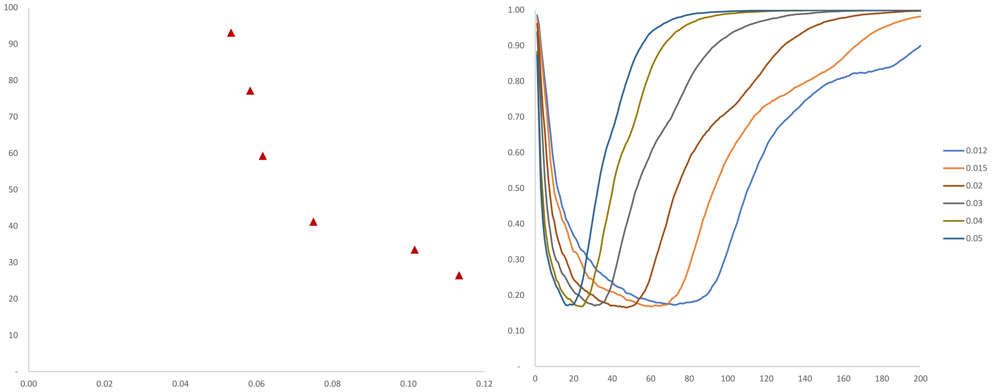


**Supplementary Figure 4.** Sensitivity to ease of migrant entry ($\beta_{\text{in}} = 2$). Left: Average time to tolerance breach ($T_\tau$) v. Total Fraction of residents ever breached ($B_f$). Right: Cell Segregation Indicator (CSI) over time.

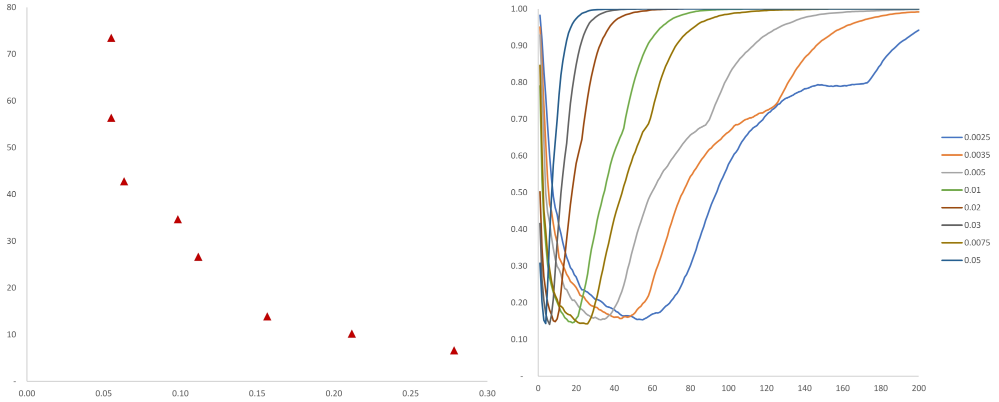


**Supplementary Figure 5.** Sensitivity to ease of migrant entry ($\beta_{\text{in}} = 0.1$). Left: Average time to tolerance breach ($T_\tau$) v. Total Fraction of residents ever breached ($B_f$). Right: Cell Segregation Indicator (CSI) over time.

**SI.4: Sensitivity to sensitivity of agent choice function:**

In our base model, an agent moves to a randomly chosen receiving neighbourhood only if it is unsatisfied with the composition of its current neighbourhood, and the receiving neighbourhood satisfies its tolerance condition. We now make the agent more sensitive to neighbourhood composition. The agent assesses the composition of a randomly chosen receiving neighbourhood and moves there if the fraction of migrants in that neighbourhood is lesser than in its current neighbourhood, irrespective of whether or not the receiving neighbourhood satisfies its threshold condition. In this case, the agent exhibits a greater sensitivity to neighbourhood composition than in our base model, where its tolerance level is the sole determinant of movement. Supplementary Table 4 presents the complete parameter set for this simulation.

**Supplementary Table 4. Model Parameters.**

| | |
|---|---|
| Number of Neighborhoods ($M$) | 20 |
| Number of Resident Agents | 1000 |
| Resident Tolerance Level ($\tau_{\text{res}}$) | 0.25 |
| Migrant Tolerance Level ($\tau_{\text{mig}}$) | 0.75 |
| Maximum population (% initial) | 150% |
| Migration Rate ($g$) | 0.005, 0.006, 0.01, 0.015, 0.02, 0.03, 0.05 |
| Number of Iterations | 200 |
| $\beta_{\text{in}}$ | 1 |

Supplementary Figure 6 (left) describes the non-linear relationship between $T_\tau$ and $B_f$, similar to the relationship observed in our based model. Supplementary Figure 6 (right) describes CSI over time for different $g$, and we see convergence of the

various trajectories as expected. It is, however, interesting to note that CSI in this case does not drop as much as in the base case before rising towards 1. This is due to the increased sensitivity in agent choice of movement, with agents now actively comparing pairs of neighbourhoods at each iteration to determine movement, rather than waiting for tolerance levels to be breached in their current neighbourhoods before attempting to move (as in the base case).

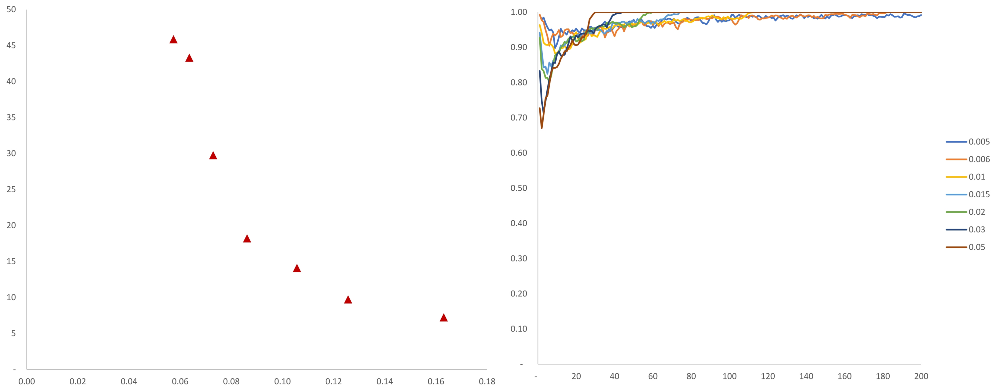


**Supplementary Figure 6.** Sensitivity to sensitivity of agent choice function. Left: Average time to tolerance breach ($T_\tau$) v. Total Fraction of residents ever breached ($B_f$). Right: Cell Segregation Indicator (CSI) over time.

Overall, our deeper exploration of the parameter space of the model appears to confirm the robustness of the findings that there is a non-linear relationship between $T_\tau$ and $B_f$, and that over the long-term segregation patterns converge irrespective of the rate of migrant entry.
